# Influences of Emotional Information on Response Inhibition in Gaming Disorder: Behavioral and ERP Evidence from Go/Nogo Task

**DOI:** 10.3390/ijerph192316264

**Published:** 2022-12-05

**Authors:** Yuzhou Chen, Hongling Yu, Xuemei Gao

**Affiliations:** 1 Faculty of Psychology, Southwest University, Chongqing 400715, China; 2 Key Laboratory of Cognition and Personality, Ministry of Education, Southwest University, Chongqing 400715, China; 3 Yucai School Huiyuan Branch Attached to Sichuan Chengdu No. 7 High School, Chengdu 610021, China

**Keywords:** gaming disorder, response inhibition, task relevance, emotional valence, ERP study

## Abstract

Background: Gaming disorder (GD) may impair executive functions such as response inhibition. According to the tripartite neurocognitive model, the interoceptive system generates a state of craving that exacerbates the dysfunction of GD. We speculate that emotional information may play an important role in the mechanism, which leads to impaired response inhibition in people with GD. Methods: A three-factor mixed experimental design was adopted in this go/nogo task. The between-subject factor was group (GD or control group), and the within-subject factors were two types of emotional information, task relevance (related or unrelated) and emotional valence (negative or positive). Results: The GD group had lower nogo accuracies than the control group in the task-unrelated condition and also in the negative condition. Parallelly, the GD group showed faster reactions and lower accuracy in the go trials than the control group under task-unrelated negative conditions. At the neural level, the GD group had smaller amplitudes of nogo-N2 and larger amplitudes of nogo-P3 than the control group in the task-unrelated condition. Conclusions: The findings prove the hypothesis of this study that emotional information could be a factor leading to impaired response inhibition in GD individuals. The response inhibition abilities of GD are weakened when processing task-unrelated or negative information, which may be caused by failure of behavioral inhibition and weakened conflict control, resulting in more cognitive resources to complete response suppression under specific conditions. This study provides evidence for weaker response inhibition in GD individuals from the perspective of cognitive–emotional interaction and provides more detailed information for interventions for GD.

## 1. Introduction

The Internet has created a new world with a vast amount of available material and the rapid transmission of information. Following this trend, we must live in a place with network coverage to study, work, and play. However, prolonged Internet use can lead to physical and mental problems. In the new International Classification of Diseases-11 (ICD-11), there are two types of Internet-based behavior listed as disorders, one is gambling disorder, and the other one, and also the most widespread, is gaming disorder (GD) [1]. In December 2020, the global prevalence of GD was estimated at 8.5% in males and 3.5% in females. Asia showed the highest prevalence of global regions (6.3%), and children and adolescents were the age group with the highest prevalence (6.6%) [2].

### 1.1. GD and Response Inhibition

GD, also known as Internet gaming disorder (IGD), is a kind of uncontrollable or persistent gaming behavior. As a behavioral addiction, GD does not require the intake of any substances, yet the condition shares psychological characteristics with substance addiction and other behavioral addictions, such as heightened arousal, craving, and tolerance [3]. Moreover, GD is associated with psychological problems such as increased aggression [4], impulsivity [5], and suicide attempts [6]. Basically, the underlying cause of these problems may be due to the impaired inhibition ability of GD individuals, resulting in uncontrollable aggressive or irrational behaviors. According to the tripartite neurocognitive model [7], GD is associated with a hypoactive “reflective” system, a hyperactive “impulsive” system, and an interoceptive system that exacerbates the dysfunction of the two systems. Regarding the reflective system, GD is manifested as reduced executive function, which appears as a decrease in response inhibition ability [8,9,10,11]. A meta-analysis found that compared with healthy individuals, individuals with GD were more likely to exhibit impaired response inhibition [12]. Once they start playing games, it is difficult to stop, even when facing severe negative consequences [13]. This result can also be reflected in the manifestation of a hyperactive impulsive system. High impulsivity may play a trigger role in engagement in gaming behaviors [14]. A recent systematic review of 32 studies found that IGD was positively associated with impulsivity [5]. Wang et al. found that people with IGD showed deficits in decision-making and tended to pursue immediate satisfaction [15]. Moreover, individuals with IGD are worse than recreational online gamers (ROGs) at risk assessment [9].

Neurologically, decreased response inhibition ability appears as abnormal amplitudes of N2 and P3. Some studies using the go/nogo task found that nogo stimuli induced larger N2 and P3 components than go stimuli [16,17]. According to previous studies, nogo-N2 is a negative wave emerging 150–400 ms after stimulation and is thought to represent conflict monitoring ability and nogo-P3 is a positive wave emerging 300–700 ms after stimulation that reflects the completion of inhibitory processing [18,19,20]. It is hypothesized that the impaired inhibition ability of Internet addicts may lead to abnormal activation of conflict monitoring and response evaluation stages, in parallel with differences in nogo-N2 and nogo-P3 amplitudes compared with normal controls [21,22,23].

### 1.2. GD and Emotion

Based on the tripartite neurocognitive model [4], the interoceptive system generates a state of craving which exacerbates the cognitive dysfunction of IGD. We speculated that psychological factors such as emotional state may play an important role in this mechanism. It is well-known that individuals with GD have more negative emotions such as depression and anxiety [24,25,26,27] and weaker emotional regulation abilities [28,29]. Through a visual search task, researchers found that individuals with Internet addiction disorder have negative attentional bias, characterized by rapid orientation towards and difficulty in releasing attention from negative emotional stimuli [30]. Another study found that the IGD group demonstrated weaker dorsal anterior cingulate cortex activation and stronger insular activation to interfering angry facial stimuli compared with the healthy control group [31]. This might reflect their dysfunction in emotion regulation, particularly in the suppression of negative emotions. To sum up, GD individuals are troubled by negative emotions. They tend to capture negative information and perceive others’ expressions as threatening, leading to an irresistible rapid orientation towards and difficulty in releasing attention from negative emotional stimuli. Therefore, the negative stimuli may dominate the processing of emotional information and impair the inhibition control system of GD individuals.

### 1.3. Emotion and Task Relevance

Task relevance is also a key factor affecting cognitive processing. Emotional stimuli associated with the task generally improve behavioral performance because additional cognitive resources are devoted to task processing; task-unrelated emotional stimuli often impair behavioral performance because unrelated information competes for cognitive resources. Previous studies have found a partial dissociation of neural activation when task-related and task-unrelated emotional information is used in an executive control paradigm [32,33,34,35,36,37]. The dual competition model hypothesizes that the impact of emotional information on executive control depends on the relevance of emotional information to the task and the threat degree of emotional information [38]. When a stimulus is unrelated to the task, impaired behavior is more likely to be observed in individuals with high anxiety levels [39]. As mentioned above, people with GD have higher levels of anxiety. Thus, we hypothesized that they may perform worse inhibition ability in task-unrelated stimuli.

### 1.4. Hypotheses

Cognition and emotion interact to determine our behaviors [40]. According to the tripartite model of GD, the psychological state could act as a catalyst to exacerbate the cognitive dysfunction of GD, but studies on response inhibition of GD mainly used neutral stimuli (e.g., letters and objects), which could not identify psychological factors affecting this process and directly determine behavioral processes in the context of social behavior. According to the above, this study adopted a three-factor experimental design, 2 (group: GD group, control group) × 2 (task: unrelated, related) × 2 (valence: negative, positive), to explore the inhibition ability of individuals with GD in response to different emotional information and speculated that the inhibition ability of GD individuals may be affected by emotional valence and task relevance. The following hypotheses will be investigated:

1. Compared to the control group, the GD group shows impaired response inhibition (i.e., lower nogo accuracy in the go/nogo tasks). This effect might be stronger when processing task-unrelated emotional information rather than task-related information.
2. Compared to the control group, the GD group shows impaired response inhibition. This effect might be stronger when processing negative emotional stimuli (i.e., angry faces) than positive emotional stimuli (i.e., happy faces).
3. Compared to the control group, the GD group shows differences in nogo-N2 and nogo-P3 amplitudes during response inhibition. This effect might be stronger when processing task-unrelated emotional information rather than task-related information.
4. Compared to the control group, the GD group shows differences in nogo-N2 and nogo-P3 amplitudes during response inhibition. This effect might be stronger when processing negative emotional stimuli rather than positive emotional stimuli.

## 2. Materials and Methods

### 2.1. Participants

This study included 145 volunteers recruited through an online questionnaire. Participants first filled out the Internet Addiction Test (IAT) and the Internet Game Disorder Questionnaire (IGD). In addition, they filled out a self-prepared online basic information questionnaire that collected general data, such as gender, age, years of education, game usage (including the top three most-played games, years of game-playing, and hours played per week), gaming percentage (the proportion of time spent playing in their online time), impulsivity, depression and anxiety levels, history of smoking, drinking, and psychiatric medication history.

For sample size, we referred to a previous study that detected the difference in inhibitory control ability between excessive social networking users and non-excessive users, applying the go/nogo paradigm and ERP techniques [23]. The study recruited 50 participants for the experiment, with 25 excessive social networking users and 25 non-excessive users. In this study, we obtained a final sample from the 145 volunteers recruited online based on the following screening criteria: the GD group included 26 participants (14 males; *M* = 20.15 years, *SD* = 1.69 years) who scored ≥ 50 points on the Internet addiction questionnaire, met ≥ 5 of the criteria in the DSM-5, and played games for >14 h per week for >1 year; the control group consisted of 24 participants (12 males; *M* = 20.17 years, *SD* = 1.44 years) who scored <50 points on the Internet addiction questionnaire, met ≤ 4 criteria in the DSM-5, and played games for <2 h per week. All participants were college students with normal vision or corrected vision and were right-handed. They provided written informed consent prior to their participation in this experiment.

### 2.2. Procedure and Tasks

The experiment was performed in a sound-attenuated, dimly lit room in the Faculty of Psychology, Southwest University, conducted by an experimenter and an assistant. Tasks were presented by E-prime 2.0 on a CRT monitor with a refresh rate of 60 Hz. Participants were asked to press (or not press) different keys on the keyboard according to the instructions wearing an EEG cap. Prior to the experiment, all participants provided written informed consent. The study used an emotional face go/nogo task. Participants were asked to identify the gender or emotional valence of faces, which was the task-unrelated and task-related processing of emotional information, respectively. Each participant completed both the unrelated and related conditions, and the order of the two tasks was balanced between participants. The experimental session lasted for 60–70 min, and the participants were compensated with 60 CNY after finishing the experiment.

#### 2.2.1. Task-Unrelated Condition

**Figure 1:**
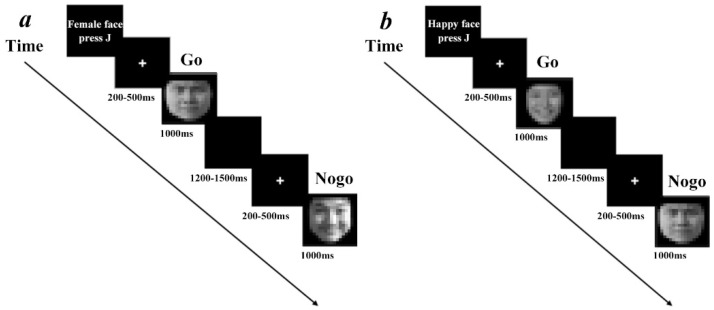
(**a**) Task-unrelated emotional face go/nogo task. (**b**) Task-related emotional face go/nogo task.

In this task, participants were asked to identify the gender of the faces and ignore their emotions; thus, the emotional information of the faces was task-unrelated. The task used the go/nogo paradigm and was divided into two blocks. In one block, participants were instructed to press a button when they saw a female face (go trial) and not to press the button when they saw a male face (nogo trial). In the other block, participants were instructed to press a button when they saw a male face (go trial) and not to press the button when they saw a female face (nogo trial). The order of the two blocks was balanced between participants. There were 240 trials per block, including 168 go trials (70%) and 72 nogo trials (30%). In each block, go and nogo trials were pseudo-random, and the go trials always preceded the nogo trials to elicit dominant motor responses and apparent conflicts during response suppression. Instructions were displayed at the beginning of each block, prompting participants to press or not press the “J” key with their right hand, depending on the gender of the face. Each trial began with a white “+” gaze point on a black screen for 200–400 ms. Subsequently, an emotional face appeared in the center of the screen for 1000 ms, participants were asked to react as quickly as possible after the picture was presented. The picture was followed by an empty black screen for 1200–1500 ms (Figure 1a).

#### 2.2.2. Task-Related Condition

In this task, participants were asked to judge the emotional valence of faces. Thus, attention was directly concentrated on the emotional information on the faces, and the emotional information was task-related while the gender information was task-unrelated. In one block, participants were instructed to press the button when they saw a happy face (go trial) and not to press it when they saw an angry face (nogo trial). In the other block, participants were instructed to press the button when they saw an angry face (go trial) and not to press it when they saw a happy face (nogo trial). The other procedures were the same as for the task-unrelated condition (Figure 1b).

### 2.3. Experimental Materials

#### 2.3.1. Emotional Face Materials

The study used 40 happy faces and 40 angry faces selected from the Chinese Facial Affective Picture System [41]; 20 male faces and 20 female faces were used in each emotional valence. Responses to the happy faces formed the positive condition in this experiment, and the responses to the angry faces formed the negative condition.

#### 2.3.2. Measures

The Internet Gaming Disorder Questionnaire [42] and the Internet Addiction Test [43] were used for grouping. The Beck Depression Inventory [44], the Self-Rating Anxiety Scale [45], and the Barratt Impulsiveness Scale [46] were used to assess levels of depression, anxiety, and impulsivity, respectively. We also used the Alcohol Use Disorder Identification Test [47] and Fagerstrom Test for Nicotine Dependence [48] to exclude participants with alcohol addiction and smoking addiction.

### 2.4. Electrophysiological Recording and Preprocessing

Brain electrical activity was recorded at 64 scalp sites using tin electrodes mounted in an elastic cap (Brain Product, Munchen, Germany), with the reference of a linked mastoid (LM). Electrodes were placed below the right eye and infra-orbitally at the left eye to record vertical electrooculogram (VEOG) and horizontal electrooculogram (HEOG), respectively. All inter-electrode impedance was maintained below 5 KΩ, and the sampling rate was 500 Hz.

The EEG data were preprocessed and analyzed using MATLAB R2014a (8.3.0.532) and the EEGLAB toolbox (v14.1.1). During the offline analysis, original EEG signals were filtered with a band-pass of 0.01 and 30 Hz. Ocular artifacts and head movements were removed from the data using the method of independent component analysis (ICA). The EEG data were segmented for each trial, spanning 200 ms prior to each picture onset to 1000 ms after the presentation of the face stimuli. The period of 200 ms pre-stimulus was used as the baseline to align the ERP amplitude. Epochs with amplitudes over ±100 μV at any site were excluded from averaging.

The time windows of different studies vary according to factors such as differences between tasks and experimental materials, types of participants, and preparatory activities [17]. For instance, time windows for nogo-N2 in previous studies have included 200–380 ms [49], 150–400 ms [18], 200–350 ms [20], and 240–300 ms [19]; for nogo-P3, time windows of previous studies include 300–500 ms [18], 350–700 ms [20], and 350–550 ms [19]. Therefore, based on the previous research and the results of this study, we chose the following time windows: the nogo-N2 was measured as the mean activity from 280 to 380 ms after stimulus onset, and the nogo-P3 was measured as the mean activity from 380 to 600 ms after stimulus onset. We selected the 14 sites where the nogo-N2 and nogo-P3 are most pronounced according to previous research [19,50]: five fronto-central sites (F3, F4, Fz, FC3, FC4), five central sites (CZ, C1, C2, C3, C4), and four centro-parietal sites (CP1, CP2, CP3, CP4).

### 2.5. Statistical Analysis

For the behavioral analysis, a three-way repeated-measures analysis of variance (ANOVA) was conducted with respect to group (two levels: GD group, control group), task (two levels: unrelated, related), and emotional valence (two levels: negative, positive). The between-subjects variable was group, the within-subjects variables were task and emotional valence, and the dependent variables were the accuracies of the nogo trials as well as the accuracies and reaction times of go trials. The accuracy of nogo trials was the percentage of correct no-response trials in the total nogo trials, and the accuracy of go trials was the percentage of correct-response trials in the total go trials. Independent sample *t*-tests were conducted to identify between-group differences.

For the ERP analysis, a four-way repeated-measures analysis of variance (ANOVA) was conducted with respect to group (GD group and control group), task (unrelated and related), emotional valence (negative and positive), and electrode point (14 sites). The dependent variables were nogo-N2 and nogo-P3 amplitudes at 14 sites, including five fronto-central sites (F3, F4, Fz, FC3, FC4), five central sites (CZ, C1, C2, C3, C4), and four centro-parietal sites (CP1, CP2, CP3, CP4). All trials used for averaged ERPs were correct trials.

SPSS Version 25.0 (IBM Corporation, Armonk, NY, USA) was used for the statistical analysis of behavioral and ERP data. Statistical analyses were adjusted for variance nonsphericity using the Greenhouse–Geisser correction. Significant interactions were analyzed by a simple effect analysis, and partial eta-squared was calculated to examine the effect size of the statistical results.

## 3. Results

### 3.1. Descriptive Results

The results (Table 1) showed that gaming years, gaming time per week, percentage of Internet use, and scores of IAT and IGD were significantly higher in the GD group than in the control group. Scores on the anxiety scale, depression scale, impulsivity scale and subscales, and alcohol use scale were also significantly higher in the GD group. Table 2 showed the descriptive results of nogo accuracies, go accuracies, go reaction times, and the mean amplitudes of nogo-N2 and nogo-P3 in two groups.

After eliminating the data of one participant with excessive artifacts, a total of 49 participants were included in the final ERP analysis, including 25 in the GD group and 24 in the control group.

### 3.2. Behavioral Results

#### 3.2.1. Response Inhibition in Nogo Trials

**Figure 2:**
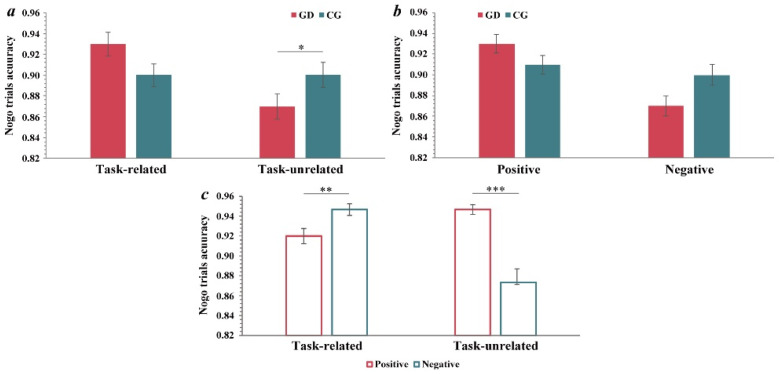
(**a**) Task × group interaction. (**b**) Valence × group interaction. (**c**) Task × valence interaction. GD = gaming disorder group, CG = control group. The error bars stand for the standard deviation of the means. * *p* < 0.05, ** *p* < 0.01, *** *p* < 0.001.

According to hypotheses 1 and 2, a three-way repeated-measures analysis of variance (ANOVA) was conducted on the accuracies of the nogo trials. The main effect of the task was significant (*F*(1, 48) = 7.60, *p* = 0.008, $\eta_{p}^{2}$ = 0.14); the accuracies in the task-related condition were significantly higher than those in the task-unrelated condition. The main effect of emotional valence was significant (*F*(1, 48) = 24.02, *p* < 0.001, $\eta_{p}^{2}$ = 0.33); the accuracies for positive emotions were significantly higher than those for negative emotions. The main effect of the group was not observed. Based on hypothesis 1, the task × group interaction was significant (*F*(1, 48) = 7.60, *p* = 0.008, $\eta_{p}^{2}$ = 0.14). Simple effect analysis showed that the accuracies of the GD group (*M* = 0.87, *SD* = 0.01) were significantly lower than those of the control group in the task-unrelated condition (*M* = 0.90, *SD* = 0.01, *p* < 0.05), no significant difference was found in the task-related condition (Figure 2a). Based on hypothesis 2, the valence × group interaction was significant (*F*(1, 48) = 11.12, *p* = 0.002, $\eta_{p}^{2}$ = 0.19). Simple effect analysis showed that the accuracies of the GD group (*M* = 0.87, *SD* = 0.01) were marginally significantly lower than those of the control group in negative condition (*M* = 0.90, *SD* = 0.01, *p* = 0.06); no significant difference was found in positive condition (Figure 2b). From another perspective, in the GD group, the accuracies for positive emotions (*M* = 0.93, *SD* = 0.01) were significantly higher than for negative emotions (*M* = 0.87, *SD* = 0.01, *p* < 0.001); in the control group, there was no significant difference. Additionally, the task × valence interaction was significant (*F*(1, 48) = 100.44, *p* < 0.001, $\eta_{p}^{2}$ = 0.68). Simple effect analysis showed that in the task-unrelated condition, the accuracies for positive emotions (*M* = 0.94, *SD* = 0.01) were significantly higher than for negative emotions (*M* = 0.83, *SD* = 0.01, *p* < 0.001). In the task-related condition, the accuracies for positive emotions (*M* = 0.90, *SD* = 0.01) were significantly lower than for negative emotions (*M* = 0.94, *SD* = 0.01, *p* = 0.003). The task × valence × group interaction was not significant, but the GD group (*M* = 0.80, *SD* = 0.02) showed lower accuracies for negative emotions than the control group (*M* = 0.85, *SD* = 0.02) only in the task-unrelated condition (*p* = 0.032, task-related condition: *p* = 0.856).

#### 3.2.2. Responses in Go Trials

**Figure 3:**
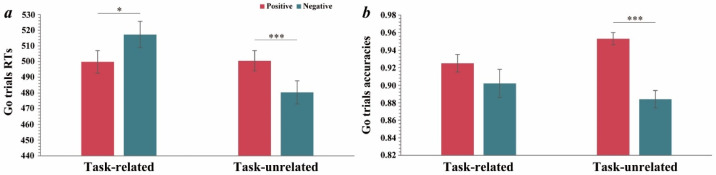
(**a**) Task × valence interaction on go trials RTs. (**b**) Task × valence interaction on go trials accuracies. The error bars stand for the standard deviation of the means. * *p* < 0.05, *** *p* < 0.001.

A three-way repeated-measures ANOVA was conducted on the go trials accuracies and reaction times (RTs) to provide explanations from another perspective. For the go trial RTs, the results showed that the main effect of the task was significant (*F*(1, 48) = 7.79, *p* = 0.008, $\eta_{p}^{2}$ = 0.14); the RTs in the task-related condition (*M* = 508.53 ms, *SD* = 6.50) were significantly longer than those in the task-unrelated condition (*M* = 490.47 ms, *SD* = 6.61). The task × valence interaction was significant (*F*(1, 48) = 17.09, *p* < 0.001, $\eta_{p}^{2}$ = 0.26). Simple effect analysis (Figure 3a) showed that in the task-unrelated condition, the RTs for positive emotions (*M* = 500.50 ms, *SD* = 6.48) were significantly higher than for negative emotions (*M* = 480.44, *SD* = 7.36, *p* < 0.001). In the task-related condition, the RTs for negative emotions (*M* = 517.23 ms, *SD* = 8.38) were significantly higher than for positive emotions (*M* = 499.84 ms, *SD* = 7.15, *p* = 0.048). The task × group interaction and valence × group interaction were not significant, as well as the three-way interaction, but we found that the RTs of the GD group (*M* = 465.39, *SD* = 10.20) were significantly shorter than those of the control group (*M* = 495.48, *SD* = 10.61, *p* = 0.046) for negative emotions in the task-unrelated condition only.

For go trial accuracies, the results showed that the main effect of the valence was significant (*F*(1, 48) = 23.22, *p* < 0.001, $\eta_{p}^{2}$ = 0.33); the accuracies for positive emotions (*M* = 0.94, *SD* = 0.01) were significantly higher than for negative emotions (*M* = 0.89, *SD* = 0.01). The task × valence interaction was significant (*F*(1, 48) = 9.51, *p* = 0.003, $\eta_{p}^{2}$ = 0.17). Simple effect analysis showed that in the task-unrelated condition (Figure 3b), the accuracies for positive emotions (*M* = 0.95, *SD* = 0.01) were significantly higher than for negative emotions (*M* = 0.88, *SD* = 0.01, *p* < 0.001). In the task-related condition, there was no significant difference. The task × group interaction, valence × group interaction, and the three-way interaction were not significant, but we found that the go accuracies of the GD group were significantly lower than those of the control group in the task-unrelated condition, both for positive emotions (*p* = 0.039) and negative emotions (*p* = 0.012). No significant difference was found in the task-related condition.

### 3.3. ERP Results

#### 3.3.1. Results of Nogo-N2

**Figure 4:**
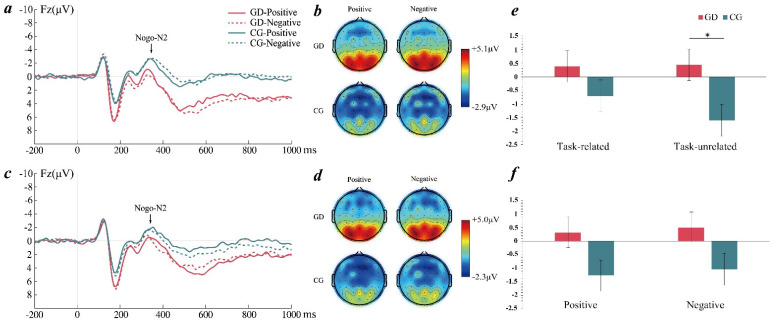
(**a**) Averaged ERPs at Fz for two groups in the task-unrelated condition; (**b**) topographic maps of nogo-N2 for two groups in the task-unrelated condition; (**c**) averaged ERPs at Fz for two groups in the task-related condition; (**d**) topographic maps of nogo-N2 for two groups in the task-related condition. GD = gaming disorder group, CG = control group. (**e**) Task × group interaction on nogo-N2 amplitudes. (**f**) Valence × group interaction on nogo-N2 amplitudes. The error bars stand for the standard deviation of the means. * *p* < 0.05.

According to hypotheses 3 and 4, a four-way repeated-measures ANOVA was conducted on the amplitudes of nogo-N2 and nogo-P3. Regarding nogo-N2, the main effect of the task was significant (*F*(1, 47) = 4.03, *p* = 0.05, $\eta_{p}^{2}$ = 0.08); the nogo-N2 amplitudes in the task-unrelated condition were significantly higher than those in the task-related condition. The main effect of the group and the valence was not significant. The main effect of the electrode point was significant (*F*(13, 35) = 6.00, *p* < 0.001, $\eta_{p}^{2}$ = 0.69); the nogo-N2 amplitudes in the central frontal region were significantly higher than those in the central apical region. Based on hypothesis 3, the task × group interaction was significant (*F*(1, 47) = 5.35, *p* = 0.025, $\eta_{p}^{2}$ = 0.10). The nogo-N2 amplitudes were significantly lower in the GD group than in the control group in the task-unrelated condition but not significantly different in the task-related condition (Figure 4). Based on hypothesis 4, the valence × group interaction was not significant (*F*(1, 47) = 0.03, *p* = 0.861, $\eta_{p}^{2}$ = 0.001). Additionally, the valence × electrode point interaction was significant (*F*(13, 611) = 2.92, *p* = 0.006, $\eta_{p}^{2}$ = 0.06). Nogo-N2 amplitudes induced by positive emotions were significantly higher than those induced by negative emotions at the CZ, C3, and CP3 positions. No other significant results were observed.

#### 3.3.2. Results of Nogo-P3

**Figure 5:**
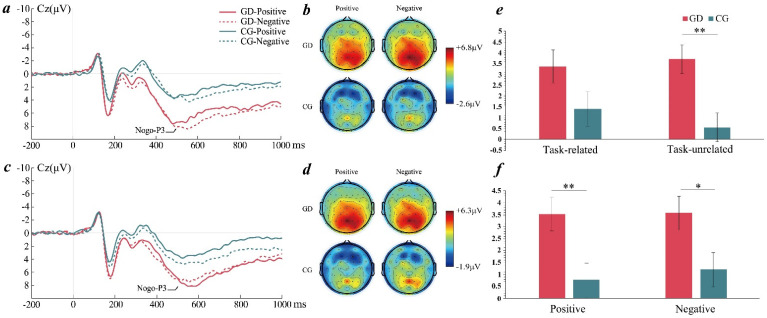
(**a**) Averaged ERPs at Cz for two groups in the task-unrelated condition; (**b**) topographic maps of nogo-P3 for two groups in the task-unrelated condition; (**c**) averaged ERPs at Cz for two groups in the task-related condition; (**d**) topographic maps of nogo-P3 for two groups in the task-related condition. GD = gaming disorder group, CG = control group. (**e**) Task × group interaction on nogo-P3 amplitudes. (**f**) Valence × group interaction on nogo-P3 amplitudes. The error bars stand for the standard deviation of the means. * *p* < 0.05, ** *p* < 0.01.

Regarding nogo-P3, the main effect of the task and the valence were not significant. The main effect of the group was significant (*F*(1, 47) = 6.75, *p* = 0.012, $\eta_{p}^{2}$ = 0.13). Nogo-P3 amplitudes were significantly higher in the GD group than in the control group. The main effect of the electrode point was significant (*F*(13, 35) = 11.62, *p* < 0.001, $\eta_{p}^{2}$ = 0.81). Nogo-P3 amplitudes were significantly higher in the central parietal region than in the central frontal region. Based on hypothesis 3, the task × group interaction was significant (*F*(1, 47) = 4.11, *p* = 0.048, $\eta_{p}^{2}$ = 0.08). Nogo-P3 amplitudes were significantly higher in the GD group than in the control group in the task-unrelated condition but not significantly different in the task-related condition (Figure 5). Based on hypothesis 4, the valence × group interaction was not significant (*F*(1, 47) = 1.08, *p* = 0.304, $\eta_{p}^{2}$ = 0.02). Additionally, the GD group had larger amplitudes of nogo-P3 with positive emotions (*M* = 0.53, *SD* = 0.70) in the task-related condition than the control group (*M* = 0.59, *SD* = 0.68, *p* = 0.038). No other significant results were observed.

## 4. Discussion

Based on the behavioral results above, task and valence are interacting factors. For the accuracies of nogo trials, participants performed better with negative faces than positive faces in the task-related condition, which may indicate an attentional bias toward negative stimuli [51,52]. The result was quite the opposite in the task-unrelated condition, where participants performed better with positive emotions than with negative emotions. The task-unrelated condition required participants to ignore the emotions of faces and judge the gender; thus the emotional information of the faces was processed as task-irrelevant information, and participants were disturbed by emotions when completing the task, especially negative ones.

### 4.1. Task Relevance and Response Inhibition in GD

In this study, the GD group performed better inhibition in the nogo trials in the task-unrelated condition than the control group. It can infer that the emotional information may impair the suppressive behaviors of GD individuals when they are task-unrelated. This phenomenon can also be explained from the perspective of the go trials: the GD group had shorter RTs in the go trials than the control group with task-unrelated negative faces and lower accuracy on both emotional valences in the task-unrelated condition, although the two effects were not very pronounced. Individuals with GD have high impulsivity characteristics according to a recent systematic review [5]. We also found that the GD group had higher impulsivity scores than the control group in the present study. The faster reactions and lower accuracy in the task-unrelated go trials may correspond to their lower nogo accuracies under the same condition.

At the neural level, previous studies have indicated that nogo-N2 is associated with the conflict monitoring of response inhibition and nogo-P3 is associated with response assessment or successful response inhibition [53,54,55,56]. In this study, the GD group showed smaller nogo-N2 amplitudes and larger nogo-P3 amplitudes than the control group when emotional information was task-unrelated, but the effect was not significant when emotional information was task-related. Previous studies have found smaller nogo-N2 amplitudes and larger nogo-P3 amplitudes in Internet addicts compared with normal controls [21,57]. The results suggested that individuals with GD had lower activation in the conflict monitoring stage and higher activation in the response assessment stage than the control group, and the effect especially existed in the task-unrelated condition. The reason for this may be that in the task-unrelated condition, participants needed to suppress the interference of emotional information while identifying the gender of the faces; while in the task-related condition, participants would not be disturbed by irrelevant emotional information. Moreover, compared with the control group, individuals with GD were more likely to be disturbed by irrelevant stimuli. Their cognitive resources for conflict monitoring and attentional control may be weakened in the task-unrelated condition, requiring more cognitive resources to successfully suppress response impulses [54,55,56], as indicated by the smaller nogo-N2 amplitudes and larger nogo-P3 amplitudes.

Various studies have found that individuals with GD exhibit impaired frontal lobe function during response inhibition which is one of the brain regions involved in the interaction between emotion and cognition [58,59,60,61,62]. The abnormalities might lead to unreasonable allocation of cognitive resources for emotional processing and response inhibition, allowing irrelevant emotional information to more easily obtain access to the processing level of the individual. Therefore, individuals with GD may show weakened nogo-N2 amplitudes and enhanced nogo-P3 amplitudes with task-unrelated information. It should also be noted that one previous study found inconsistent results with our study, manifested as enhanced nogo-N2 and reduced nogo-P3 amplitudes in excessive social networking site (SNS) users compared with non-excessive users [22]. The reason could be that the study used addiction-related stimuli (SNS logos), while the present study used task-unrelated stimuli (face images), and so did other studies (letters, numbers) [21,56]. The addiction-related stimuli are more likely to attract the attention of Internet addicts, and therefore may lead to enhanced activation of conflict monitoring and reduced activation of response evaluation.

### 4.2. Emotional Valence and Response Inhibition in GD

The effect of emotional valence was also one of the findings of this study. Studies have reported that the human brain has a processing bias towards negative events compared with neutral and positive stimuli and that threat-related stimuli can more effectively capture attention [63,64,65,66,67,68,69]. However, the more quickly one focuses on negative stimuli, the more difficult it is to disengage from them [70,71], especially for people with high levels of negative emotions. In this study, we found that the nogo accuracies for positive stimuli were significantly higher than for negative stimuli in the GD group, the effect was not significant in the control group. The dual competition model indicates that emotional stimuli with higher threat levels will reduce behavior performance, and behavior performance impairment is more frequently observed in individuals with high anxiety levels [38]. Previous research has demonstrated that individuals with GD have more negative emotions, such as depression and anxiety [23,24,25,26]. In this study, the self-reported depression and anxiety levels were also significantly higher in the GD group than in the control group. The negative emotions may make it difficult for individuals with GD to disengage from the information, thereby impairing their inhibition ability.

In this study, the valence × group interaction was not significant in the ERP results. The GD group and the control group did not show significant differences in nogo-N2 amplitudes in both negative and positive stimuli, but the two groups showed significant differences in nogo-P3 amplitudes in both negative and positive stimuli. The reason could be that many studies have proven the effect of emotional valence on response inhibition using the go/nogo paradigm, but most of them selected emotional valence as the only within-subject variable [72,73,74,75,76]. That means the emotional valence was only processed in the task-related condition in their studies. The valence of faces relies more on visual perception which requires conscious intervention, while valence in the task-unrelated condition may not fully show its effect. This study combining emotional valences and task levels in one paradigm, therefore, would affect the effects of emotional valence in statistical analysis. The results of this study proved our speculation. Although the valence × group interaction was significant in the behavioral results, we found that it was only significant in the task-related condition (*p* = 0.005, $\eta_{p}^{2}$ = 0.153), but not significant in the unrelated condition (*p* = 0.097, $\eta_{p}^{2}$ = 0.056). We also found the valence × group ERP results had a more significant tendency in the task-related condition than in the unrelated condition.

### 4.3. Contributions and Limitations

Given the results described above, this study provides behavioral and neural evidence for impaired response inhibition in individuals with GD under conditions of different emotional information. Our study was based on the tripartite neurocognitive model to study the effect of emotion on the cognitive dysfunction of GD and proved that negative or task-unrelated emotion could be a critical factor of impaired inhibition ability in individuals with GD. The results enrich the interaction theory between cognition and emotion in GD and provide evidence for the connection between the interoceptive system and executive systems in the tripartite model of GD. Moreover, this study provides a theoretical basis for GD intervention. Interventions should attach importance to not only behavior modification, but also the regulation of psychological factors, such as the principle of cognitive behavioral therapy (CBT).

Despite the strengths, this investigation was also characterized by some limitations. First, some of the results in our study were significant but not very pronounced, or marginally significant. The relatively small sample size may be the reason for the inadequate statistical power to detect potential behavioral and ERP differences. Thus, larger samples may be necessary for future research. Second, the participants in this study were recruited through a self-selected online procedure, which may be a bias to study altered online behaviors such as GD. Future studies could recruit clinically diagnosed participants if conditions permit. Third, this study did not control the gaming condition of participants prior to the experiment. Studies have found that playing internet games 48 h before an experiment affects the results [77,78]. Future studies should consider the gaming condition prior to the experiment as a control variable. Fourth, faces are just one possible emotional cue, and response inhibition appears to be an index of many potential processes, such as expression recognition. Future studies should add a measure of emotional engagement, such as an autonomic marker or self-reported trial-by-trial ratings. Last, this study only compared positive and negative faces and did not include neutral faces. Future investigations should include neutral faces, and the selection of negative faces could be expanded to include faces indicating fear or disgust.

## 5. Conclusions

This study explored the response inhibition and neural mechanisms of GD at different emotional levels. The findings showed that the response inhibition of individuals with GD was regulated by task relevance and emotional valence. The response inhibition ability of individuals with GD was weakened when processing task-unrelated or negative information. This effect was manifested as more impulsive responses, failures of behavioral inhibition, and weakened conflict control for individuals with GD, leading to more consumption of cognitive resources to complete response suppression. This study provided evidence for the impaired response inhibition of GD individuals from the perspective of the interaction theory between cognition and emotion and provided more detailed information for interventions for GD.

## Figures and Tables

**Table 1:** Demographic characteristics of the gaming disorder group and control group.

	GD	CG	*t*	*p*
Age	20.15 (1.69)	20.17 (1.44)	−0.03	0.822
Education years	14.19 (1.02)	13.88 (1.39)	0.92	0.155
Gaming years	4.65 (2.73)	1.71 (1.82)	4.45 *	0.040
Gaming time per week (h)	23.75 (8.02)	1.40 (0.88)	13.57 ***	0.000
Gaming percentage (%)	58.38 (26.48)	17.17 (17.38)	6.50 *	0.027
IAT	67.08 (9.08)	35.17 (8.39)	12.88 ***	0.000
IGD	6.62 (1.24)	1.17 (1.34)	14.96 ***	0.000
BDI	17.35 (11.76)	10.04 (8.68)	2.48 *	0.017
SAS	46.96 (9.37)	42.67 (5.14)	1.99 *	0.011
BIS-Total	43.17 (11.52)	33.82 (7.56)	3.42 ***	0.001
BIS-Cognitive	25.73 (4.05)	23.42 (3.74)	2.10 *	0.041
BIS-Motor	30.12 (6.67)	26.50 (4.25)	2.30 *	0.028
BIS-Planning	25.96 (5.33)	20.67 (4.30)	3.84 ***	0.000
AUDIT	3.77 (4.78)	1.04 (1.78)	2.63 ***	0.000
FTND	0.73 (2.15)	0.50 (1.69)	0.42	0.400

Notes: Means and standard deviations (in parentheses) of demographic characteristics. CG = control group, GD = gaming disorder group. *t* = value of Student’s *t* test, *p* = value of possibility. Gaming percentage = the proportion of time spent playing games in their online time. IAT = the Internet Addiction Test. IGD = the Internet Game Disorder Questionnaire. BDI = the Beck Depression Inventory. SAS = the Self-Rating Anxiety Scale. BIS = the Barratt Impulsiveness Scale (BIS-11), the scale has three subscales (cognitive impulsiveness, motor impulsiveness, and assessing impulsive planning). AUDIT = the Alcohol Use Disorder Identification Test. FTND = the Fagerstrom Test for Nicotine Dependence. * *p* < 0.05, *** *p* < 0.001.

**Table 2:** Behavioral and EEG descriptive results in different groups.

	GD (*n* = 25)				CG (*n* = 24)			
	Task-Unrelated		Task-Related		Task-Unrelated		Task-Related	
	Positive	Negative	Positive	Negative	Positive	Negative	Positive	Negative
Behavior								
Nogo-ACC	0.93 (0.07)	0.80 (0.09)	0.93 (0.05)	0.93 (0.09)	0.95 (0.03)	0.85 (0.07)	0.87 (0.10)	0.94 (0.04)
Go-ACC	0.94 (0.07)	0.86 (0.09)	0.93 (0.08)	0.89 (0.13)	0.97 (0.03)	0.91 (0.05)	0.92 (0.06)	0.91 (0.10)
Go-RT	493.58 (44.32)	467.68 (54.16)	489.36 (45.40)	513.41 (64.46)	508.91 (47.62)	495.48 (49.31)	507.70 (54.64)	518.34 (52.70)
ERP								
Nogo-N2	0.31 (0.57)	0.58 (0.61)	0.35 (0.62)	0.42 (0.59)	−1.72 (0.58)	−1.52 (0.62)	−0.84 (0.63)	−0.58 (0.60)
Nogo-P3	3.61 (0.68)	3.82 (0.67)	3.43 (0.79)	3.31 (0.80)	0.53 (0.70)	0.59 (0.68)	1.01 (0.81)	1.81 (0.82)

Notes: Means and standard deviations (in parentheses) of the results. GD = gaming disorder group, CG = control group. ACC = accuracy, RT (ms) = reaction time. ERP shows the mean amplitudes of nogo-N2 and nogo-P3.

## Data Availability

The data used to support the findings of this study are available from the corresponding author upon request.
